# Supplementary material for: Epidemiology of West Nile virus in Africa: An underestimated threat
Source: PLoS Negl Trop Dis. 2022 Jan 10;16(1):e0010075. doi: 10.1371/journal.pntd.0010075 (PMC8789169; doi:10.1371/journal.pntd.0010075)
Supplement: Attachment — Submitted filename: review WNV epidemiology in Africa.doc [file pntd.0010075.s002.doc]

**'Epidemiology of West Nile virus in Africa: an underestimated threat'**

(PLOS Neglected Tropical Diseases, ms. PNTD-D-21-01059)

*A review of the manuscript*

This is a comprehensive and useful synopsis.

Remarks and suggestions:

Line 79: ... such as birds, corvids and raptors... corvids and raptors are birds.

Line 189: *Ae. aegypti* is really a recognized vector of WNV?

Line 204 (Tab.1): “Isolation” here means the virus isolation or also virus RNA molecular detection? Be more specific and differentiate, please.

Line 207 (Tab.2): Infection rate 14·28% means 14.28% (decimal point) or 14-28% ? (and several similar cases). “Virus titer”: inoculated or recovered titer? Explain, please.

Line 222 (Tab.3): The (unusual) cases of WNV transmission by *Ornithodoros savignyi* and *Argas arboreus* ticks need to be decribed (commented) in detail.

Line 237 (Tab.4): “Antibodies detection”: describe the serological test used (whether VNT, PRNT, HIT, CFT or ELISA), and a differentiation from other African flaviviruses (if done). This is (serology data) a weak point of the whole synopsis; flaviviruses are namely serologically readily cross-reactive and some serological results without a differentiation among related flaviviruses could thus be erroneous.

Line 237 (Tab.4): “Viral detection” means the virus isolation or viral RNA detection? Be more specific and differentiate, please.

Line 249 (Tab.5): “Viral isolation” means the virus isolation or viral RNA detection? Be more specific and differentiate, please.

Line 249 (Tab.5): “Antibody detection” and “Seroprevalence rate” describe the serological tests used, and differentiation from other African flaviviruses, if done.

Line 255 (Tab.6): “Antibody detection” and “Viral detection” – see above.

Line 255 (Tab.6): “Experimental infection” + means disease, antibodies or only attempt without any signs?

Line 262: outbreaks

Line 267: possibly a redundant sentence.

Line 269: Tunisia is already mentioned on the line 264.

Line 276 (Tab.7): “Seroprevalence rate” – see above.

Line 318: why you do not quote original paper of Hungarian record on WNV-2 ?

Line 326-331: are all these “isolations” from so many mosquito species really WNV isolations, or also WNV RNA molecular detections? Check it, please, and note.
