# Supplementary material for: Epidemiology of West Nile virus in Africa: An underestimated threat
Source: PLoS Negl Trop Dis. 2022 Jan 10;16(1):e0010075. doi: 10.1371/journal.pntd.0010075 (PMC8789169; doi:10.1371/journal.pntd.0010075)
Supplement: Attachment — Submitted filename: Response to reviewers.docx [file pntd.0010075.s003.docx]

**Reviewers major comments:**

**Reviewer #1:**

- ***Methods***

**Methods used for particular serological surveys must be notified.**

We thank the reviewer for raising this important point. Methods used for serological surveys have been specified in Tables 4 – 7.

- ***Results***

**Serological methods used for serosurveys data need to be notified, and possible cross-reactions with other African flaviviruses described, if possible. Modification is also required for "virology" data (virus isolation or RNA molecular detection).**

We thank the reviewer for raising this important point and we apologize for the lack of clarity.

Methods used for serological surveys have been specified in Tables 4 – 7. Virus isolation or RNA molecular detection has been specified in Tables 1,3,4,5, and 6.

In addition, two new paragraphs, dedicated to the results obtained by serosurveys and the possible bias due to possible cross-reactions, have been added. One paragraph was added to Discussion (Lines 482– 498), as follows:

“Several WNV seroprevalence studies carried out on humans and animals have been reported, providing evidence of an intense WNV circulation in the Continent. In particular, as illustrated by Figure 2 and reported by the studies of Table 4, 5, 6 and 7, seropositivity in human to WNV has been reported in Morocco, Tunisia, Algeria, Egypt, Mali, Senegal, Sierra Leone, Ivory Coast, Ghana, Cameroon, Nigeria, Chad, Sudan, South Sudan, Djibouti, Ethiopia, Kenya, Central African Republic, Rwanda, Uganda, Zambia, Namibia, Botswana, South Africa, and Madagascar. Diverse serological methods have been used in different countries, ranging from HIT, ELISA, VNT, micro-VNT, PRNT, MIA, CFT, IFA, WB, and EIA. For 19 out of 99 serological studies (18.81%) [42, 62, 64, 71, 76, 83, 87, 88, 91, 93, 105, 107, 110, 120, 123, 128, 136, 142, and 146] only ELISA test was used without a specific WNV neutralization test, and therefore the potential of cross-reactivity with closely related pathogens, such as the Usutu virus, the St. Louis encephalitis virus, or the Japanese Encephalitis virus cannot be excluded [86]. Finally, detailed information on the serological tests used are not available for 18 of these studies (17.82%) [15,18,35,45,46,50,70,77,85,86,112,113,116,126,131,138,147, and 148]. It would be extremely important, in the next future surveys, to carry out WNV confirmation tests with standard methods as the micro-VNT or PRNT, in order to obtain specific serological responses, particularly in areas characterized by circulation of several Flaviviruses [120]. Unfortunately, many times the nature, the remaining volume, and the storage procedures of the samples, do not allow to do so. Despite these limitations, these serologic findings associated with viral isolation through cell culture and RNA molecular detection in mosquitoes, few ticks, birds, horses, humans, and other mammals indicate that WNV is actively circulating in many areas of the African continent.”

And another one was added to Conclusions (Lines 512-521), as follows:

“The actual epidemiological situation in most countries of the African continent is unknown, due to: i) the non-specificity of clinical signs of WNV infection in respect to other arboviruses, very often indistinguishable from each other and from other tropical diseases such malaria, typhoid fever or undifferentiated febrile illness [7,125]; ii) the poor information management and sharing of public health data system of most African countries [150]; iii**) the possible bias obtained by serological analysis due to the utilization of non-specific WNV neutralization assays, where the risk of cross-reactivity among closely related pathogens cannot be excluded**, and iv) the restricted availability of diagnostic capacity, lack of awareness and inconsistent surveillance, with most investigations performed only during outbreak periods [7,73,124]. All these factors make quantification of the yearly WNV circulation at continental level difficult, with only few accurate data available in North and Sub-Saharan Africa.”

- ***Conclusions***

**Conclusions are well formulated, except for serosurveys.**

We thank the reviewer for the appreciation of our work. Serosurveys descriptions are added to the main text within discussion and conclusions.

**Reviewer #2:**

- ***Methods***

**The article is a literature review that follows PRISMA and QUORUM criteria (referenced). The approach is appropriate, and the authors have identified a considerable number of articles that are relevant to the study.**

**The authors have made contact with experts (n = 29) to obtain further information although what this consists of is not explained or the identity of these individuals.**

**No statistical analysis was conducted on the review.**

**There are not ethical or regulatory concerns on this manuscript.**

We thank the reviewer for the description of our work. We revised our manuscript taking into account the important and helpful reviewer’s comments, which we believed greatly improved our study.

N = 29 are the number of articles suggested by experts. With “experts” we refer to the manuscript co-authors and other experts working at Institut Pasteur de Dakar, Fondazione Edmund Mach and OIE Reference Laboratory for WND (Istituto Zooprofilattico Sperimentale dell’Abruzzo e Molise).

After revision, following co-authors’ and Reviewer’s suggestions, the number of articles suggested by experts became 33.

- ***Results***

**The analysis does reflect the aims of the review. The authors have compiled a considerable volume of information concerning the epidemiology of West Nile virus in Africa presented in a series of seven tables and cite 150 references.** **However, a better description of West Nile virus phylogeny should be presented in lines 81/82 to explain what lineages 3,4,5,6 & 7 are, their relationship to the main lineages 1 and 2 and why they have not been detected in Africa, where presumably WNV evolved.**

**The authors should clarify on lines 135 to 136 that only lineage 1 emerged in the Americas.**

**The assertion on lines 143 to 144 that because an isolate from Tunisia roots the LI isolates from North America suggests "a possible flow between Tunisia and the Americas" ignores the presence of an isolate within that clade from Israel (1998).** **This needs to be revised to suggest that phylogeny suggest that viruses circulating in the Middle East / North Africa are related to those in North America.**

**The lines 198 to 202 are not explained very well and do not critically assess the papers they cite. They need to define EIP, state the small number of mosquitoes that were infected in reference 21 and if these species are poorly competent for WNV, how does the virus persist?**

**The discussion on line 218 on tick transmission of WNV is highly speculative.**

**A general comment is that the authors have documented a large volume of data but this is not matched by the discussion in the text, which could be improved.**

We thank the reviewer for raising these important points. We revised our manuscript taking into account the important and helpful reviewer’s comments, which we believed greatly improved our study.

After revision, following co-authors and reviewer’s suggestions, the number of references became 157: original articles have been added to the bibliography and articles suggested by co-authors incorporated.

See below a point-by-point response to the comments raised by the reviewer.

**However, a better description of West Nile virus phylogeny should be presented in lines 81/82 to explain what lineages 3,4,5,6 & 7 are, their relationship to the main lineages 1 and 2 and why they have not been detected in Africa, where presumably WNV evolved**

Following the reviewer’s comments, a better description of West Nile virus phylogeny to explain what lineages 3,4,5,6 & 7 are, their relationship to the main lineages 1 and 2 and why they have not been detected in Africa, where presumably WNV evolved, has been introduced at lines 87-107, as follows:

“WNV currently includes up to nine phylogenetic lineages, identified through phylogenetic analyses: WNV lineage 1 (WNV-L1) to lineage 9 (WNV-L9) [29]. WNV-L7 has been recently classified as a distinct flavivirus, the Koutango virus (KOUTV) [2,15]. Among all these observed lineages, only WNV-L1, L2, and L8, other than the KOUTV, have been detected in Africa [2]. WNV-L1 and L2 are the most important for the Public Health point of view because they are most pathogenic and widespread, and implicated in several outbreaks worldwide [3,7]. WNV-L1, mainly diffused in Central and Northern Africa, emerged in Europe in the 1960s [3]. After 30 years of silent circulation, it started causing epidemics in Northern Americas, Northern African, Western, and Eastern European countries [3]. The main actor of the European scenario was WNV-L1 up to 2004 when WNV-L2, considered endemic in Southern Africa and Madagascar, was reported for the first time in Hungary [3]. Since 2010 it started causing several outbreaks in central Europe and it is nowadays one of the main responsible of WNV infections in Europe [3,7].

Other less widespread lineages are WNV-L3, also known as Rabensburg virus, present in southern Moravia and Czech Republic; WNV-L4, isolated and reported in Russia; WNV-L5, isolated in India and often considered as the clade 1c of WNV-L1, and WNV-L6, based on a small gene fragment, isolated in Spain [1,2]. Finally, putative lineage 9, often considered a sub lineage of WNV-L4, has been isolated from Uranotaenia unguiculata mosquitoes in Austria [29]. These lineages, never isolated in Africa, might have evolved from distinct introductions into the Northern Hemisphere [25].

**The authors should clarify on lines 135 to 136 that only lineage 1 emerged in the Americas.**

We clarified (lines 159 – 162) that only lineage 1 emerged in the Americas in 1999, as follows:

“WNV-L1 was successfully introduced into Europe in the 1960s while it appeared for the first time in North America in 1999, subsequently becoming endemic across both continents [1]. WNV-L2, after its first appearance in Hungary in 2004, showed multiple introductions into Europe [1,2,16].

**The assertion on lines 143 to 144 that because an isolate from Tunisia roots the LI isolates from North America suggests "a possible flow between Tunisia and the Americas" ignores the presence of an isolate within that clade from Israel (1998). This needs to be revised to suggest that phylogeny suggest that viruses circulating in the Middle East / North Africa are related to those in North America.**

This part (lines 168-175) has been revised, suggesting that viruses circulating in the Middle East / North Africa are related to those in North America, as follows:

“Interestingly, phylogenetic analysis demonstrated that the strain *PaH001* isolated in Tunisia in 1997 roots the tree of WNV-L1 circulating in North America [12,20]. Furthermore, phylogenetic and genetic distance studies evidenced that the Tunisian strain *PaH001* is closely related to a group of highly conserved viruses collected in America and Israel between 1998 and 2000, suggesting that viruses circulating in the Middle East / North Africa are related to those circulating in North America [12].

**The lines 198 to 202 are not explained very well and do not critically assess the papers they cite. They need to define EIP, state the small number of mosquitoes that were infected in reference 21 and if these species are poorly competent for WNV, how does the virus persist?**

A better explanation on *Cx. neavei* and *Cx. quinquefasciatus* vector competent experiments has been given (lines 246-261), as follows, critically assessing the papers they cited:

“*Cx. neavei*, attracted by both horses and birds, and widespread in different type of lands, might have a possible role as bridge vectors in the sylvatic transmission [21]. These mosquito species have been shown to be more efficient vectors for WNV-L1 than L8, and to be susceptible to WNV-L2 and KOUTV infections [21]. However, WNV transmission has not been observed for WNV-L2 and KOUTV [21]. As shown in Table 2, experimental infections conducted on *Cx. neavei* in South Africa [35,156] showed high transmission rate but a 50% infection threshold, observed after exposing birds with different viraemia to this mosquito species, of 4.4 logs per ml, that was higher than those observed in *Cx. univittatus* mosquitoes (2.1 logs per ml) [156]. Furthermore, the time taken by *Cx. neavei* and *Cx. quinquefasciatus* mosquitoes to develop WNV after infection (extrinsic incubation period, EIP) has been estimated to last 15 days at 27°C [21]. However, the infective life survival rate has been estimated to be comprised between 0.75 and 0.88 for *Cx. neavei* and between 0.87 and 0.88 for *Cx. quinquefasciatus* mosquitoes [21]. Based on these data, only 1.3% to 10.4% of *Cx. neavei* and 12.59% to 15.45% of *Cx. quinquefasciatus* mosquitoes have been estimated to survive at 15 days post infection by the authors of the study [21]. Despite *Cx. quinquefasciatus* and *Cx. neavei* mosquitoes being widely distributed in the African continent and their proven competence for distinct lineages of WNV, such findings imply a low probability of WNV transmission to new hosts in the African continent and they might indicate a low impact of *Cx. neavei* and *Cx. quinquefasciatus* mosquitoes in WNV circulation in Africa [21]. However, the small number of mosquitoes tested, as reported in reference 21, and the lack of experimental infection using different temperature would require further studies.”

**The discussion on line 218 on tick transmission of WNV is highly speculative.**

This part (lines 299 – 305) has been revised, as follows, and we believe is now improved:

“A large number of ticks are carried around the world by mammals and by birds during their migration paths [56]. Further studies are needed to assess the possible contribution of ticks on the maintenance, transmission, and spread of WNV over long distances and extensive periods of time. Considering that WNV competent hosts are characterized by relatively short viremic duration periods, the role of ticks as WNV vectors should be further evaluated especially for better explaining the biological mechanism which favors virus translocation from the African continent into the others [6,60].”

**A general comment is that the authors have documented a large volume of data but this is not matched by the discussion in the text, which could be improved.**

Discussion (lines 403-506) has been revised and we believe is now improved.

- ***Conclusions***

**This subject is neglected and more investigations of West Nile virus epidemiology are needed. However, as the authors state, biological science in many African countries (of which there are 54 recognized by the United Nations) are under-resourced and diagnostics for the virus limited. It then seems odd to then advocate for a widespread and long-term WNV surveillance system for the continent when there is no in-country activity. Who would provide the coordination to achieve this and who would fund it?**

**The authors mention climate change on line 363, seems a bit late to include this in the final sentences.**

We thank the reviewer for raising these important points. We revised our manuscript taking into account the important and helpful reviewer’s comments.

See below a point-by-point response to the comments raised by the reviewer.

**This subject is neglected and more investigations of West Nile virus epidemiology are needed. However, as the authors state, biological science in many African countries (of which there are 54 recognized by the United Nations) are under-resourced and diagnostics for the virus limited. It then seems odd to then advocate for a widespread and long-term WNV surveillance system for the continent when there is no in-country activity. Who would provide the coordination to achieve this and who would fund it?**

We thank the reviewer for raising these important points.

We agree with the reviewer that most of African countries are under-resourced, and diagnostic of the virus limited. However, we strongly believe it is important to highlight the need of a national surveillance plan implementation for monitoring WNV in areas at risk of viral introduction and circulation and the possible spread across continents. A West Nile virus surveillance can be established in Africa based on the national surveillance program of the Yellow Fever virus, set up in the African continent by the ministries of health in partnership with the WHO to better understand the dynamics of circulation of this neglected pathogen.

Conclusions (lines 532-548) have been revised, as follows, and we believe is now improved:

“New surveillance strategies for preventing and control WNV in Africa need to be implemented not only to better assess the current health impact but also to prevent and control future outbreaks, and therefore limit transmission of the disease. National and district levels should cooperate with Ministries of Health, and other international partners such as the WHO and European public and veterinary health bodies, to implement national surveillance programs in African countries and coordinates surveillance action between Africa and Europe. These programs core objectives should i) minimize the suffering and damage, ii) prevent national and international spread, and iii) contain outbreaks through strong surveillance for early detection and rapid response, as done before for other viruses, as the Yellow Fever and Poliomyelitis viruses.

The ability to accurately identify pathogens in a timely and accurate way has grown in the last few decades, and it has become increasingly clear that the implementation of a collaborative international and multi-disciplinary One Health action, based on the analyses of the interconnection among environmental , humans’ and animals’ health factors in different Continents, will be crucial to allow a more accurate risk assessment and thus an early response to West Nile virus but also to other emerging zoonotic pathogens by public and veterinary health actors.”

**The authors mention climate change on line 363, seems a bit late to include this in the final sentences.**

We removed the sentence at lines 525-527. We agree it is inappropriate to refer to climate change in the final sentences.

**Reviewer #3**

- ***Methods***

**This is a review article. The methods of publication selection is clearly described.**

We thank the reviewer for the appreciation of our work.

- ***Results***

**The results extracted from the literature has been summarized very well and are presented in a concise way.**

We thank the reviewer for the appreciation of our work.

- ***Conclusions***

**The conclusions are supported by the various publications, which have been thoroughly analyzed by the authors.**

We thank the reviewer for the appreciation of our work.

**Minor revision:**

**#Reviewer 1**

**Line 79: ... such as birds, corvids and raptors... corvids and raptors are birds.**

We apologize for the lack of clarity. The sentence has been modified from line 85, as follows:

“Among birds, corvids and raptors appear highly susceptible to WNV infection, resulting in higher incidence with severe neurological signs that lead the individuals to the death [9,10].”

**Line 189: Ae. aegypti is really a recognized vector of WNV?**

We thank the reviewer for raising this important point and we apologize for the incorrect information.

It was not right to include *Ae. aegypti* as vector competent mosquito for WNV in Africa.

Thanks to the reviewer’s comment we realize that a wrong citation was accidentally introduced in the text: that was the reference no. 36. The reference “An updated checklist of mosquito species (Diptera: Culicidae) from Madagascar. Parasite Paris Fr. 2016” has been now substituted with “Review of West Nile virus circulation and outbreak risk in Madagascar: Entomological and ornithological perspectives. Parasite. 2016; 23:49”.

*Ae. aegypti* vector competent experiments have never been done in Africa on WNV.

We got confused by citation n. 36 (Review of West Nile virus circulation and outbreak risk in Madagascar: Entomological and ornithological perspectives), where the authors state:

“*The virus has been isolated from* Aedes aegypti *mosquitoes, which is present in tropical and subtropical locations as well and are a potential threat for transmission of WNV to humans. Vector competence has been demonstrated in other countries for* Aedes aegypti, Ae. albopictus, Culex univittatus, Cx. quinquefasciatus, Cx. pipiens, Cx. vansomereni, Mansonia africana, *and* Ma. Uniformis”.

Although in this paper authors discuss about WNV *Ae. aegypti* vector competence studies, these studies have never been conducted in the African continent. Despite *Ae. aegypti* mosquitoes resulted competent with an African strain and WNV has been isolated in Madagascar in this species (Table 1), it might be speculative to affirm that *Ae. aegypti* is a WNV vector competent mosquito in Africa, given the fact that no studies have been carried out in this sense in the Continent.

This information has been eliminated also from Table 2 (this study has not been conducted in Africa)

| **Country** | **Days post infection** | **Infection rate** | **Transmission rate** | **Dissemination rate** | **Species [lineage; inoculated virus titer]** | **References** |
| --- | --- | --- | --- | --- | --- | --- |
| Madagascar | (∙∙) | 16% | < 16% | (∙∙) | *Aedes aegypti* | [36] |

Interestingly, the study of Turell, 2001 demonstrated the WNV vector competence of *Ae. aegypti* in the USA. This information is added at lines 475 – 477:

“However, laboratory studies conducted in the USA demonstrated the vector competence of local *Ae. aegypti* strains for WNV, although the species was found to be less efficient than *Cx. pipiens* mosquitoes [153]”.

In Africa, *Ae. aegypti* has been demonstrated to be Koutango virus (nowadays considered a distinct flavivirus) competent vector:

- [https://digitalcommons.lsu.edu/cgi/viewcontent.cgi?article=3084&context=gradschool_dissertations](about:blank)
- Ref 152 : Jean Coz, G.L.G., Michel Cornet, Michel Valade, M. O. Lemoine, A. Gueye, Transmission experimentale d'un arbovirus du groupe B le virus Koutango par Aedes aegypti L. Cah. O.R.S.T.O.M., ser. Entomology Medica et Parasitology, 1975. XIII (N.2): p. 57-62.
- Ref 15: Fall G, Diallo D, Soumaila H, Ndiaye EH, Lagare A, Sadio BD, et al. First Detection of the West Nile Virus Koutango Lineage in Sandflies in Niger. Pathog Basel Switz. 2021; 10(3): 257.

The text has been modified from line 262 to line 264, explaining *Ae. Aegypti* vectorial competence for the KOUTV, as follows:

“Interestingly, KOUTV viral dissemination and vertical transmission has been observed in *Ae. aegypti* mosquitoes in Senegal [15,151,152] while WNV vector competence experiments have never been conducted in this mosquito species in the African continent.”

In addition, Table 1 and 2, have also been updated, as well as Discussion (lines 473-477), as follows:

“Particular attention should be paid towards *Ae. aegypti* mosquitoes in Africa. Although WNV has been isolated in wild specimens belonging to this species [51], which was also found to be capable of KOUTV transmission in Senegal [21,151,152], *Ae. aegypti* African populations have never been tested for WNV vectorial competence so far. However, laboratory studies conducted in the USA demonstrated the vector competence of local *Ae. aegypti* strains for WNV, although the species was found to be less efficient than *Cx. pipiens* mosquitoes [153].

To give a more comprehensive information on WNV recognized vectors, a new revised text has been added from lines 216-266, as follows:

“In Africa, the most widespread WNV vectors mainly belong to the *Culex pipiens* complex (*Cx. pipiens* s.l.) [7,30-32] which includes *Cx. p. pipiens* Linnaeus, *Cx. p. pipiens* biotype molestus Forskal, *Cx. p. quinquefasciatus* Say, *Cx. p. pallens* and *Cx.* *torrentium* Martini [33,34]. As summarized in Table 1, WNV has been isolated from at least 46 mosquito species but studies to assess their vectorial competence have been carried out between 1972 and 2016 only in 8 mosquito species (*Cx. quinquefasciatus, Cx. univittatus**, Cx. vansomereni Edwards, Ma. uniformis, Ma. Africana, Cx. pipiens, Cx. theileri,* and *Cx.* *neavei*) in Madagascar, the Maghreb region (Algeria, Morocco, Tunisia), Senegal, Kenya, and South Africa (Table 2) [18,21,22,26,27,30,35-50,155]. These studies provide evidence of the vector competence of *Cx. pipiens*, *Cx. quinquefasciatus,* *Cx. vansomereni, Cx. univittatus*, *Cx. theileri*, and *Cx. neavei* mosquitoes in Africa [21,30,34-36,49,51].

*Cx. pipiens*, characterized by high dissemination and transmission rates, are indicated by numerous reports as the most important vector species of WNV [30,32,35,37,42,43,46,49,50].

High ornithophilic and low anthropophilic *Cx. univittatus* mosquitoes are considered both highly susceptible and efficient transmitters of WNV [35]. WNV vertical transmission has been described under field condition in *Cx. univittatus* males in the Rift Valley province of Kenya [39].

*Cx. theileri* is a less efficient WNV vector: despite being highly susceptible to the virus it has a lower transmission rate [35,157]. Vector competent experiments highlight that this mosquito species can be infected as much as *Cx. univittatus*, but probably due to its host preferences (less ornithophilic than *Cx. Univittatus* and feeding mostly at ground level) there are very few WNV isolates made from wild population, classifying this species as a poor vector [157].

*Cx. quinquefasciatus*, shown to be a competent species for WNV-L1 and -L8, is widespread in urban environments and active all year-round, and might be considered as another important WNV vector, especially in urban contexts [21]. Furthermore, vector competence studies highlighted that *Cx. quinquefasciatus* mosquitoes were not competent for KOUTV [21].

*Cx. neavei*, attracted by both horses and birds, and widespread in different type of lands, might have a possible role as bridge vectors in the sylvatic transmission [21]. These mosquito species have been shown to be more efficient vectors for WNV-L1 than L8, and to be susceptible to WNV-L2 and KOUTV infections [21]. However, WNV transmission has not been observed for WNV-L2 and KOUTV [21]. As shown in Table 2, experimental infections conducted on *Cx. neavei* in South Africa [35,156] showed high transmission rate but a 50% infection threshold, observed after exposing birds with different viraemias to this mosquito species, of 4.4 logs per ml, that was higher than those observed in *Cx. univittatus* mosquitoes (2.1 logs per ml) [156]. Furthermore, the time taken by *Cx. neavei* and *Cx. quinquefasciatus* mosquitoes to develop WNV after infection (extrinsic incubation period, EIP) has been estimated to last 15 days at 27°C [21]. However, the infective life survival rate has been estimated to be comprised between 0.75 and 0.88 for *Cx. neavei* and between 0.87 and 0.88 for *Cx. quinquefasciatus* mosquitoes [21]. Based on these data, only 1.3% to 10.4% of *Cx. neavei* and 12.59% to 15.45% of *Cx. quinquefasciatus* mosquitoes have been estimated to survive at 15 days post infection by the authors of the study [21]. Despite *Cx. quinquefasciatus* and *Cx. neavei* mosquitoes being widely distributed in the African continent and their proven competence for distinct lineages of WNV, such findings imply a low probability of WNV transmission to new hosts in the African continent and they might indicate a low impact of *Cx. neavei* and *Cx. quinquefasciatus* mosquitoes in WNV circulation in Africa [21]. However, the small number of mosquitoes tested, as reported in reference 21, and the lack of experimental infection using different temperature would require further studies.

Interestingly, KOUTV viral dissemination and vertical transmission has been observed in *Ae. aegypti* mosquitoes in Senegal [15,151,152] while WNV vector competence experiments have never been conducted on this mosquito species in the African continent.

The ability of different mosquito species to transmit specific lineages of WNV highlights a direct correlation between vector competence and genetic variability [21].”

With a detailed check of the bibliography, we realized that vector competent experiments have also been conducted in *Cx. vansomereni* Edwards (reference no.36), and additional reports on WNV isolation in mosquitoes in Africa have been found out (reference no. 64). These findings have been added to Table 1, Table 2, and to the text.

Following this consideration, text at line 220 “As summarized in Table 1, WNV has been isolated from at least 42 mosquito species” has been modified where the number 42 has been substituted with the number 46.

This information has been added to Table 1 and to the chapter “West Nile virus vectors in Africa” (lines 220 – 230), as follows:

“As summarized in **Table 1**, WNV has been isolated from at least 46 mosquito species but studies to assess their vectorial competence have been carried out between 1972 and 2016 only in 8 mosquito species (*Cx. quinquefasciatus*, *Cx. univittatus*, *Cx. vansomereni Edwards, Ma. uniformis*, *Ma. Africana*, *Cx. pipiens*, Cx. *theileri*, and *Cx. neavei*) in Madagascar, the Maghreb region (Algeria, Morocco, Tunisia), Senegal, Kenya, and South Africa (**Table 2**) [18,21,22,26,27,30,35-50,155]. These studies provide evidence of the vector competence of *Cx. pipiens, Cx. quinquefasciatus*, *Cx. univittatus,* *Cx. theileri*, and *Cx. neavei* mosquitoes in Africa [21,30,34-36,49,51].

and to the discussion (Line 447 – 469), as follows:

“In Africa, WNV isolation on cell cultures and RNA molecular detection have been obtained from 46 mosquito species, as shown in Table 1. Among them, *An. brunnipes, An. coustani, An. maculipalpis, An. pauliani, An. scotti, An. spp., Aedeomyia africana, Aedeomyia madagascarica, Ae. albocephalus, Ae. (Aedimorphus) dalzieli, Ae. juppi + caballus, Ae. vexans, Ae. madagascariensis, Ae. albothorax, Ae. circumluteolus, Ae. aegypti, Ae. africanus, Ae. Tritaeniorhynchus, Ae. spp., Cx. antennatus, Cx. decens group, Cx. ethiopicus, Cx. guiarti, Cx. nigripes, Cx. perfuscus, Cx. perexiguus, Cx. poicilipes, Cx. pruina, Cx. quinquefasciatus, Cx. scottii, Cx. spp., Cx. weschei, Coquillettidia metallica, Coquillettidia microannulata, Coquillettidia richiardii, Ma. africana, Ma. uniformis, Mi. hispida, Mi. lacustris, and Mi. splendens* have never been tested for their WNV vector competence (Table 2) and therefore they should be the future subject for WNV laboratory vector competence experiments.

Vector competence experiments conducted in Madagascar, Algeria, Morocco, Tunisia, Senegal, Kenya, and South Africa highlight the vector competence of *Cx. pipiens, Cx. quinquefasciatus*, *Cx. univittatus,* *Cx. theileri*, *Cx. vansomereni,* and *Cx. neavei* mosquitoes in Africa [21,30,34-36,49,51,155].”

With a detailed check of the bibliography, we realized that vector competent experiments have been conducted on *Cx. tritaeniorhynchus* mosquitoes in Pakistan, but not in Africa, as shown by Ref “Hayes CG, Basit A, Bagar S, Akhter R. Vector competence of Culex tritaeniorhynchus (Diptera: Culicidae) for West Nile virus. J Med Entomol. 1980 Mar 31;17(2):172-7. doi: 10.1093/jmedent/17.2.172. PMID: 6988591”. This information has been erased from Table 3 and from the text (line 222, line 225).

**Line 204 (Tab.1): “Isolation” here means the virus isolation or also virus RNA molecular detection? Be more specific and differentiate, please.**

We thank the reviewer for raising this important point and we apologize for the lack of clarity.

Following the reviewer’s suggestions, Table 1 has been modified. In the column “References” the following apical letters have been added to each reference:

^a^ = “virus isolation on cell cultures / injection into suckling mice. Viruses detected by immunofluorescence assay using specific mouse immune ascitic fluids”;

^b^ = “RNA molecular detection”;

^c^ = “viral isolation, not specified if a and/or b”.

**Line 207 (Tab.2): Infection rate 14·28% means 14.28% (decimal point) or 14-28%? (and several similar cases). “Virus titer”: inoculated or recovered titer? Explain, please.**

We apologize for the lack of clarity. With 14·28% we mean 14.28% (decimal point) (in all similar cases).

“Virus titer”: inoculated. Table 2 has been modified accordingly.

Table 2: Original papers have been double checked and new information regarding vector competent experiments conducted in Kenya on *Cx. Vansomereni, Ma. africana, Cx. quinquefasciatus, Cx. univittatus, Ma uniformis*, and on Cx. theileri in South Africa has been added to Table 2.

Table 2 legend has been modified, as follows:

“Infection rate (number of infected mosquito bodies per 100 mosquitoes tested); Dissemination rate (number of mosquitoes with infected legs/wings per 100 mosquitoes infected); Transmission rate (number of mosquitoes with infected saliva per 100 mosquitoes with infected legs/wings); Transmission rate^a^: % of orally exposed mosquitoes (regardless of their infection status) that took a second bloodmeal and transmitted virus by bite (no. feeding); Transmission rate^b^: % of orally exposed mosquitoes with a disseminated infection that took a second bloodmeal and transmitted virus by bite (no. feeding); Transmission rate^c^: % inoculated mosquitoes that transmitted virus by bite (no. feeding); Transmission rate^d^: % of all mosquitoes with a disseminated infection (inoculated orally exposed) that transmitted virus by bite (no. feeding); (∙∙) Not defined; [ ] Lineage and inoculated virus titer (PFU/mL); n.t = not tested; *18 – 20 days after infective feed; ** 14-15 days after infective feed.”

In table 2, information related to vector competent experiments conducted on *Ae. aegypti* has been eliminated, because not conducted in Africa but in the USA Ref: “Turell M, O’Guinn ML, Dohm DJ, Jones JM. Vector competence of North American mosquitoes (Diptera: Culicidae) for West Nile virus. Journal of Medical Entomology. 2001; 38, 130–134.”

**Line 222 (Tab.3): The (unusual) cases of WNV transmission by Ornithodoros savignyi and Argas arboreus ticks need to be described (commented) in detail.**

We agree with the reviewer that the unusual cases of WNV transmission by *Ornithodoros savignyi* and *Argas arboreus* ticks need to be described in detail. Following this consideration, a detailed description has been added to the text (lines 290-298), as follows:

“Particular attention should be given to *Ornithodoros savignyi* and *Argas arboreus* ticks as potential vectors of WNV in the African continent. In Egypt, experimental infections of adult soft tick *O. savignyi*, using a local strain of WNV (Ar-248), showed that the species got infected without being competent. However, after paternal infection, *O. savignyi* could transmit the virus to infant mice and WNV isolation could be obtained from its coxal fluid. [52].

*Argas arboreus* ticks are also shown to be WNV competent vectors and vertical and horizontal transmission has been observed for this species. The virus titer was detected to be 10^4 PFU/mL at 4 days post infection (pi), remaining constant at 10^3 PFU/mL from day 6 to day 50pi. After 20 days from experimental infections, *A. arboreus* adults could transmit the virus to uninfected chickens. Furthermore, F1 *A. arboreus* larvae from WNV experimentally infected females could also transmit the virus to uninfected chickens. WNV was isolated from *A. arboreus* salivary glands, synganglia, and coxal fluids [54].”

Despite the recent classification of the Koutango virus (KOUTV) as a distinct flavivirus, the virus has been for long time considered a distinct lineage of WNV (Lineage 7). For this reason, we also consider important to add to Table 3 information related to KOUTV isolation on *Rhipicephalus muhsamae* in Senegal, *Hyalomma marginatum rufipes*, and *Ripicephalus guilhoni* ticks in Senegal.

Table 3 was modified. In the column “References” the following apical letters have been added to each reference:

^a^ = “virus isolation on cell cultures / injection into suckling mice. Viruses detected by immunofluorescence assay using specific mouse immune ascitic fluids”;

^b^ = “RNA molecular detection”;

^c^ = “viral isolation, not specified if a and/or b”; reference numbers with no apical letters only refer to experimental infections.

This specification is reported in the table legend.

**Line 237 (Tab.4): “Antibodies detection”: describe the serological test used (whether VNT, PRNT, HIT, CFT or ELISA), and a differentiation from other African flaviviruses (if done). This is (serology data) a weak point of the whole synopsis; flaviviruses are namely serologically readily cross-reactive and some serological results without a differentiation among related flaviviruses could thus be erroneous.**

We thank the reviewer for raising this important point and we apologize for the lack of clarity.

Under the reviewer suggestions Table 4 has been modified. In the column “References” the following apical letters have been added to each reference:

^d^ = “hemagglutination-inhibition test (HIT)”;

^e^ = “serological surveys, type of antibodies detection tests non specified”;

^f^ = “epitope-blocking enzyme-linked immunosorbent assay (ELISA)”;

^g^ = “virus-neutralization test (VNT)”;

^h^ = “micro virus-neutralization test (micro-VNT)”;

^i^ = “immunoglobulin M (IgM)-specific ELISA”;

^l^ = “plaque reduction neutralization test (PRNT)”;

^m^ = “Flavivirus microsphere immunoassay (MIA)”.

This specification is reported in the table legend.

Each study reported in Table 4 have been double checked, and we realized that WNV has been detected in Zimbabwe in *Riparia paludicola* and *Hirundo rustica* birds through RNA molecular detection but not through serological studies. This information has been corrected in the text (erased from the list of serological studies reported in line 321 and line 464, added to the list of countries with no seroprevalence studies, line 400), in Table 4 and Figure 2.

**Line 237 (Tab.4): “Viral detection” means the virus isolation or viral RNA detection? Be more specific and differentiate, please.**

We thank the reviewer for raising this important point and we apologize for the lack of clarity.

Specific information about viral isolation, meaning the virus isolation through cellular culture or also virus RNA molecular detection has been added to Table 4.

In particular, to references of Table 4 we added the apical letters:

^a^ = “virus isolation on cell cultures / injection into suckling mice. Viruses detected by immunofluorescence assay using specific mouse immune ascitic fluids”;

^b^ = “RNA molecular detection”;

^c^ = “viral isolation, not specified if a and/or b”; reference numbers with no apical letters only refer to experimental infections.

This specification is reported in the table legend.

**Line 249 (Tab.5): “Viral isolation” means the virus isolation or viral RNA detection? Be more specific and differentiate, please.**

Specific information about viral isolation, meaning the virus isolation through cellular culture or also virus RNA molecular detection has been added to Table 5.

In particular, to references of Table 5 we added the apical letters:

^a^ = “virus isolation on cell cultures / injection into suckling mice. Viruses detected by immunofluorescence assay using specific mouse immune ascitic fluids”;

^b^ = “RNA molecular detection”;

^c^ = “viral isolation, not specified if a and/or b”.

This specification is reported in the table legend.

**Line 249 (Tab.5): “Antibody detection” and “Seroprevalence rate” describe the serological tests used, and differentiation from other African flaviviruses, if done.**

We thank the reviewer for raising this important point and we apologize for the lack of clarity.

Under the reviewer suggestions Table 5 has been modified, adding to the references the following apical notes:

^d^ = “hemagglutination-inhibition test (HIT)”;

^e^ = “serological surveys, type of antibodies detection tests non specified”;

^f^ = “epitope-blocking enzyme-linked immunosorbent assay (ELISA)”;

^g^ = “virus-neutralization test (VNT)”;

^h^ = “micro virus-neutralization test (micro-VNT)”;

^i^ = “immunoglobulin M (IgM)-specific ELISA”;

^l^ = “plaque reduction neutralization test (PRNT)”;

^m^ = “flavivirus microsphere immunoassay (MIA)”;

^n^ = “immunoblotting method (WB)”.

**Line 255 (Tab.6): “Antibody detection” and “Viral detection” – see above.**

We thank the reviewer for raising this important point and we apologize for the lack of clarity.

Under the reviewer suggestions Table 6 has been modified, adding to the references the following apical notes:

^a^ = “virus isolation on cell cultures / injection into suckling mice. Viruses detected by immunofluorescence assay using specific mouse immune ascitic fluids”;

^b^ = “RNA molecular detection”;

^c^ = “viral isolation, not specified if a and/or b”; antibodies detection via

^d^ = “hemagglutination-inhibition test (HIT)”;

^e^ = “serological surveys, type of antibodies detection tests non specified”;

^f^ = “epitope-blocking enzyme-linked immunosorbent assay (ELISA)”;

^g^ = “virus-neutralization test (VNT)”;

^h^ = “micro virus-neutralization test (micro-VNT)”;

^i^ = “immunoglobulin M (IgM)-specific ELISA”;

^l^ = “plaque reduction neutralization test (PRNT)”;

^m^ = “flavivirus microsphere immunoassay (MIA)”;

^n^ = “complement fixation test (CFT)”;

“Experimental infection” means ^o^ = “disease”, ^p^ = “antibodies” or ^q^ = “attempt without any clinical signs/development of viraemia”; ^r^ = “case of illness”.

All references reported in table 6 have been carefully checked and some corrections have been made:

- African forest buffalo (Syncerus caffer) and domestic cuttle (*Bos taurus*): RNA molecular detection, South Africa [91] has been added to the table

- Fallow deer (*Dama dama*): RNA molecular detection, South Africa [91] has been added to the table. No antibodies detection experiments have been made for this species.

- Domestic dog (*Canis lupus familiaris*): RNA molecular detection and case of illness, South Africa [18,91] have been added to the table.

- Giraffe *(Giraffa*): RNA molecular detection, South Africa [91] has been added to the table. No antibodies detection experiments have been made for this species.

- Goat (*Capra aegagrus hircus*): RNA molecular detection, South Africa [91] has been added to the table.

- Humped camel (*Camelus bactrianus*): Reference 97 has been erased from the table because not pertinent.

- Lemur (*Galago senegalensis*): RNA molecular detection, Senegal [93] has been added to the table. No antibodies detection experiments have been made for this species.

- Lion (*Panthera leo*): RNA molecular detection, South Africa [91] has been added to the table. No antibodies detection experiments have been made for this species.

- Livestock, South Africa: RNA molecular detection has been added to the table. RNA molecular detection has been performed on livestock with acute fever, neurologic disease, or other signs of acute infection in South Africa [74].

- Oxen (*Bos*): The paper n.92 has been substituted in the bibliography by a new paper (see comment referred to line 318). The correct papers for this study are papers no .7 and no. 154

- Pigs (*Sus*): Experimental infection without any developed sign, South Africa. Reference no. 7 and 93.

Reference 35 has been removed from the cited references in the table because not pertinent.

- Roan antelope (Hippotragus equinus): RNA molecular detection has been added to the table [91]. No antibodies detection experiments have been made for this species.

- Small rodents (*Rodentia*): RNA molecular detection has been added to the table [101].

- Sheep (*Ovis aries*): Reference 95 has been added to the table, reference 100 has been erased because not pertinent.

- White rhinoceros (*Ceratotherium simum*): added to table, according to serological studies highlighted in reference 91.

**Line 255 (Tab.6): “Experimental infection” + means disease, antibodies or only attempt without any signs?**

We apologize for the lack of clarity. In Table 6 (Lines 366-367) we added the following explanation:

“Experimental infection” means ^o^ = “disease”, ^p^ = “antibodies” or ^q^ = “attempt without any clinical signs/development of viraemia”; reference numbers with no apical letters only refer to case of illness.

In addition to Table 6 we added:

- Domestic dog: viral detection / case of illness: [18] The dog had severe diarrhea, became comatose, had convulsions, and died. The dog was initially thought to have rabies, and subsequently a flavivirus isolated from brain tissue was thought to be WESSV, but ultimately was shown to be WNV.

- Small rodents: [101] Viral isolation in Nigeria

- Goat: [91] viral isolation

**Line 262: outbreaks**

Done (After corrections line 262 became line 374)

**Line 267: possibly a redundant sentence.**

We thank the reviewer for letting us notice the presence of a redundancy. The sentence has been removed (line 379-380).

**Line 269: Tunisia is already mentioned on the line 264.**

We thank the reviewer for letting us notice the presence of a redundancy. Tunisia has been removed from line 382.

**Line 276 (Tab.7): “Seroprevalence rate” – see above.**

We apologize for the lack of clarity.

Following reviewer’s suggestions Table 7 has been modified, adding to the references the following apical notes:

“antibodies detection via: ^d^ = “hemagglutination-inhibition test (HIT)”; ^e^ = “serological surveys, type of antibodies detection tests non specified”; ^f^ = “epitope-blocking enzyme-linked immunosorbent assay (ELISA)”; ^g^ = “virus-neutralization test (VNT)”; ^h^ = “micro virus-neutralization test (micro-VNT)”; ^i^ = “immunoglobulin M (IgM)-specific ELISA”; ^l^ = “microtiter serum neutralization (SN) test”; ^m^ = “plaque reduction neutralization test (PRNT)”; ^n^ = “flavivirus microsphere immunoassay (MIA)”; ^o^ = “complement fixation test (CFT)”.

**Line 318: why you do not quote original paper of Hungarian record on WNV-2?**

We thank the reviewer for the suggestion. We agree that it should be the good reference to quote.

Following reviewer’s suggestion, we added the original paper, corresponding now to reference 92. Previous reference 92 became reference 154.

**Line 326-331: are all these “isolations” from so many mosquito species really WNV isolations, or also WNV RNA molecular detections? Check it, please, and note.**

We thank the reviewer for raising this important point and we apologize for the lack of clarity.

We added to the table 1 the following reference notes:

^a^ = “virus isolation on cell cultures / injection into suckling mice. Viruses detected by immunofluorescence assay using specific mouse immune ascitic fluids”;

^b^ = “RNA molecular detection;”

^c^ = “viral isolation, not specified if a and/or b”

In the text, the meaning of WNV isolations has been clarified (lines 447-448), as follows:

“In Africa, WNV isolation on cell cultures and RNA molecular detection have been obtained from 46 mosquito species, as shown in Table 1.”

**Presented serosurveys data in this synopsis are the weak point of the manuscript; flaviviruses are namely serologically readily cross-reactive and** **some "positive" serological results without a differentiation among related flaviviruses could thus be erroneous (in certain cases it might be unclear whether specific antibodies against West Nile virus were really detected).**

A paragraph regarding the results obtained by serosurveys and the possible bias due to cross-reactions have been added to the discussion (lines 482 – 498), as follows:

“Several WNV seroprevalence studies carried out on humans and animals have been reported, providing evidence of an intense WNV circulation in the Continent. In particular, as illustrated by Figure 2 and reported by the studies of Table 4, 5, 6 and 7, seropositivity in human to WNV has been reported in Morocco, Tunisia, Algeria, Egypt, Mali, Senegal, Sierra Leone, Ivory Coast, Ghana, Cameroon, Nigeria, Chad, Sudan, South Sudan, Djibouti, Ethiopia, Kenya, Central African Republic, Rwanda, Uganda, Zambia, Namibia, Botswana, South Africa, and Madagascar. Diverse serological methods have been used in different countries, ranging from HIT, ELISA, VNT, micro-VNT, PRNT, MIA, CFT, IFA, WB, and EIA. For 19 out of 99 serological studies (18.81%) [42, 62, 64, 71, 76, 83, 87, 88, 91, 93, 105, 107, 110, 120, 123, 128, 136, 142, and 146] only ELISA test was used without a specific WNV neutralization test, and therefore the potential of cross-reactivity with closely related pathogens, such as the Usutu virus, the St. Louis encephalitis virus, or the Japanese Encephalitis virus cannot be excluded [86]. Finally, detailed information on the serological tests used are not available for 18 of these studies (17.82%) [15,18,35,45,46,50,70,77,85,86,112,113,116,126,131,138,147, and 148]. It would be extremely important, in the next future surveys, to carry out WNV confirmation tests with standard methods as the micro-VNT or PRNT, in order to obtain specific serological responses, particularly in areas characterized by circulation of several Flaviviruses [120]. Unfortunately, many times the nature, the remaining volume, and the storage procedures of the samples, do not allow to do so. Despite these limitations, these serologic findings associated with viral isolation through cell culture and RNA molecular detection in mosquitoes, few ticks, birds, horses, humans, and other mammals indicate that WNV is actively circulating in many areas of the African continent.”

and to Conclusions (Lines 512-521), as follows:

“The actual epidemiological situation in most countries of the African continent is unknown, due to: i) the non-specificity of clinical signs of WNV infection in respect to other arboviruses, very often indistinguishable from each other and from other tropical diseases such malaria, typhoid fever or undifferentiated febrile illness [7,125]; ii) the poor information management and sharing of public health data system of most African countries [150]; iii) **t****he possible bias obtained by serological analysis due to the utilization of non-specific WNV neutralization assays, where the risk of cross-reactivity among closely related pathogens cannot be excluded,** and iv) the restricted availability of diagnostic capacity, lack of awareness and inconsistent surveillance, with most investigations performed only during outbreak periods [7,73,124]. All these factors make quantification of the yearly WNV circulation at continental level difficult, with only few accurate data available in North and Sub-Saharan Africa.”

**Reviewer #2:**

**The general format of the paper is satisfactory although a few minor changes are needed.
Lines 81 to 98, its unclear why there are six 1/2 sentence paragraphs. These need to be re-written into larger sections. The final words of the introduction are a word for word repeat of the abstract, revise. Line 326, why are all these mosquito species listed, surely just refer to one of the tables?**

We thank the reviewer for appreciating our work. We revised our manuscript taking into account the important and helpful reviewer’s comments, which we believed greatly improved our study.

See below a point-by-point response to the minor comments raised by the reviewer.

**Lines 81 to 98, its unclear why there are six 1/2 sentence paragraphs. These need to be re-written into larger sections.**

Lines 87 – 115 have been re-written into larger sections, as follows:

“WNV currently includes up to nine phylogenetic lineages, identified through phylogenetic analyses: WNV lineage 1 (WNV-L1) to lineage 9 (WNV-L9) [29]. WNV-L7 has been recently classified as a distinct flavivirus, the Koutango virus (KOUTV) [2,15]. Among all these observed lineages, only WNV-L1, L2, and L8, other than the KOUTV, have been detected in Africa [2]. WNV-L1 and L2 are the most important for the Public Health point of view because they are most pathogenic and widespread, and implicated in several outbreaks worldwide [3,7]. WNV-L1, mainly diffused in Central and Northern Africa, emerged in Europe in the 1960s [3]. After 30 years of silence, it started causing epidemics in Northern Americas, Northern African, Western, and Eastern European countries [3]. The main actor of the European scenario was WNV-L1 up to 2004 when WNV-L2, considered endemic in Southern Africa and Madagascar, was reported for the first time in Hungary [3]. Since 2010 it started causing several outbreaks in central Europe and it is nowadays one of the main responsible of WNV infections in Europe [3,7].

Other less widespread lineages are WNV-L3, also known as Rabensburg virus, present in southern Moravia and Czech Republic; WNV-L4, isolated and reported in Russia; WNV-L5, isolated in India and often considered as the clade 1c of WNV-L1, and WNV-L6, based on a small gene fragment, isolated in Spain [1,2]. Finally, putative lineage 9, often considered a sub lineage of WNV-L4, has been isolated from *Uranotaenia unguiculata* mosquitoes in Austria [29]. These lineages, never isolated in Africa, might have evolved from distinct introductions into the Northern Hemisphere [25].

Translocation of diverse WNV lineages from the original ecological niches to new geographic areas is generally thought to occurs mainly through migratory birds, although the final chain of events that lead to the introduction or reintroduction of the virus into new continent needs further explanations [7]. For example, phylogenetic analyses revealed that all European WNV-L1 and 2 strains are derived from a limited number of initial independent introductions, most likely directly from Africa, followed by local amplification and spread [12,13].

WNV current distribution, genetic variability, ecology, and epidemiological pattern in the African continent is only partially known despite the general consensus on the urgency to obtain such information for quantifying the actual disease burden in Africa other than to predict future threats at continental and global scale.”

**The final words of the introduction are a word for word repeat of the abstract, revise.**

The final part of the abstract has been modified (Lines 37-41) as follows:

“This study provides the state of art on WNV investigation carried out in Africa, highlighting several knowledge gaps regarding i) the partial knowledge on the current WNV distribution and genetic diversity, ii) its ecology and transmission chains including the role of different arthropods and vertebrate species as competent reservoirs, and iii) the real disease burden for humans and animals. This review highlights the needs for further research and coordinated surveillance efforts on WNV in Africa.”

The final part of the author summary has been modified (Lines 54-61) as follows:

“However, this review highlights also the lack of knowledge regarding the occurrence and intensity of circulation of WNV in many African countries. Therefore, considering the sensitivity of WNV transmission system to climate and other environmental changes, along with the increasing level of interconnections among continents due to globalization, the intensification of the research activities on WNV and a promotion of a coordinated surveillance actions across African and European countries would provide the information of utility for a better evaluation of the actual risk of WNV and disease burden at trans-continental scale”

**Line 326, why are all these mosquito species listed, surely just refer to one of the tables?**

The list of mosquito species refers to Table 1. We now made it explicit in the text (Lines 447 – 459), as follows:

“In Africa, WNV isolation on cell cultures and RNA molecular detection have been obtained from 46 mosquito species, as shown in Table 1. Among them, *An. brunnipes, An. coustani, An. maculipalpis, An. pauliani, An. scotti, An. spp., Aedeomyia africana, Aedeomyia madagascarica, Ae. albocephalus, Ae. (Aedimorphus) dalzieli, Ae. juppi + caballus, Ae. vexans, Ae. madagascariensis, Ae. albothorax, Ae. circumluteolus, Ae. aegypti, Ae. africanus, Ae. spp., Cx. Tritaeniorhynchus, Cx. antennatus, Cx. decens group, Cx. ethiopicus, Cx. guiarti, Cx. nigripes, Cx. perfuscus, Cx. perexiguus, Cx. poicilipes, Cx. pruina, Cx. quinquefasciatus, Cx. scottii, Cx. spp., Cx. weschei, Coquillettidia metallica, Coquillettidia microannulata, Coquillettidia richiardii, Ma. africana, Ma. uniformis, Mi. hispida, Mi. lacustris, and Mi. splendens* have never been tested for their WNV vector competence (Table 2) and therefore they should be the future subject for WNV laboratory vector competence experiments.”

**The manuscript "Epidemiology of West Nile virus in Africa: an underestimated threat" provides a comprehensive review of West Nile virus epidemiology in Africa. This is a neglected subject and worth discussing. As described in previous section, the manuscript could be improved, and the authors should be more critical of the subject and in their discussion in the text. The conclusions should also be of more practical benefit.**

We revised our manuscript taking into account the important and helpful reviewer’s comments, which we believe that greatly improved our study. We think we obtained a more critical synopsis and a better formulated discussion in the text.

Conclusions have also been revised (Lines 532 – 548), as follows:

“New surveillance strategies for preventing and control WNV in Africa need to be implemented not only to better assess the current health impact but also to prevent and control future outbreaks, and therefore limit transmission of the disease. National and district levels should cooperate with Ministries of Health, and other international partners such as the WHO and European public and veterinary health bodies, to implement national surveillance programs in African countries and coordinates surveillance action between Africa and Europe. These programs core objectives should i) minimize the suffering and damage, ii) prevent national and international spread, and iii) contain outbreaks through strong surveillance for early detection and rapid response, as done before for other viruses, as the Yellow Fever and Poliomyelitis viruses.

The ability to accurately identify pathogens in a timely and accurate way has grown in the last few decades, and it has become increasingly clear that the implementation of a collaborative international and multi-disciplinary One Health action, based on the analyses of the interconnection among environmental , humans’ and animals’ health factors in different Continents, will be crucial to allow a more accurate risk assessment and thus an early response to West Nile virus but also to other emerging zoonotic pathogens by public and veterinary health actors.”

**Reviewer #3:**

**The only minor comment I have is:
Line 81: "WNV currently includes eight phylogenetic lineages [11]. Among all these observed eight lineages, only ...". Please change this sentence to: "WNV currently includes UP TO NINE phylogenetic lineages [11]. Among THESE LINEAGES, only ...". Instead of ref. [11] I suggest to use here ref. [29] (Pachler et al.).**

We thank the reviewer for raising this important point. We revised the sentence (lines 87-89)

which now reads as follows:

“WNV currently includes up to nine phylogenetic lineages, identified through phylogenetic analyses: WNV lineage 1 (WNV-L1) to lineage 9 (WNV-L9) [29]. WNV-L7 has been recently classified as a distinct flavivirus, the Koutango virus (KOUTV) [2,15].”

**This article is a nice review of the West Nile virus epidemiology in Africa. It summarizes in a clear and concise way the topic.**

We thank the reviewer for the appreciation of our work.
