# Supplementary material for: Epidemiology of West Nile virus in Africa: An underestimated threat
Source: PLoS Negl Trop Dis. 2022 Jan 10;16(1):e0010075. doi: 10.1371/journal.pntd.0010075 (PMC8789169; doi:10.1371/journal.pntd.0010075)
Supplement: Attachment — Submitted filename: Response to reviewers.docx [file pntd.0010075.s004.docx]

**Reviewers minor comments:**

**Reviewer #2: The authors have adequately addressed all the reviewers comments. The only issue now is that the text in places is unclear and could be improved throughout.**

We thank the reviewer for the appreciation of our work. We revised our manuscript taking into account the important and helpful reviewer’s comments, which helped at improving the readability of our study.

See below a list of corrections:

Line 84, no need to capitalize public health.

This sentence has been revised (Line 83).

Line 86, correct to North America

This sentence has been revised (Line 85).

Line 89, ".. one of the main lineages responsible for WNV..."

This sentence has been revised (Line 88).

Line 91, South Moravia is a part of the Czech Republic, rephrase the sentence.

This sentence has been rephrased (Line 90).

Line 98, continents

This sentence has been revised; the word “continent” has been changed into its plural form (Line 97).

Line 134, correct to " We identified 408 articles. After duplicates were removed, the remaining 395 records were screened by title, abstract and full text."

This sentence has been rephrased, following the reviewer suggestions (Line 133).

Line 140, "WNV is a biologically diverse virus..."

The word “very” has been erased, following the reviewer suggestion (Line 139).

Line 142, "...to be between the..."

This sentence has been revised (Line 141).

Line 161, "WNV-L2 was considered for a long time to be less pathogenic..."

This sentence has been revised (Line 160).

Line 170, "..only cluster 3 occurs in Africa.."

This sentence has been revised (Line 169).

Line 333, Section 4 deals with the distribution of WNV in Africa rather than epidemiology, suggest change the title.

The title of section 4 has been changed to: “West Nile virus distribution in humans” (Line 331).

Line 383, "..1960s, re-emerged.."

This sentence has been revised (Line 381).

Line 390, "..countries, causing disease among horses..." (horse can't describe symptoms?)

This sentence has been revised, including a description of symptoms in horses. In particular, line 387-390: “WNV-L2, for long time believed to be restricted to Sub-Saharan Africa and considered not pathogenic, is nowadays endemic and the most prevalent in several African and European countries, provoking clinical symptoms, mainly neurologic signs of infection including ataxia, weakness, recumbence, seizures, and muscle fasciculation, among horses in South Africa [7,74], and pathogenesis among horses, humans and birds in Europe [3].”

Line 402, Still not clear why all these species are listed. Suggest replace with "Many of these mosquito species have not been tested for their competence for WNV...."

This sentence has been rephrased, following the reviewer suggestions (Line 401).

Line 422, what is "paternal infection"?

The word “paternal” has been corrected to: “parenteral” (Line 415).

Line 433, overuse of "the" delete them all and it reads better.

Numerous “the” at line 425 and 426 have been erased, following reviewer suggestion

Line 436, ".. such as micro-VNT.."

The sentence at line 429 has been revised

Line 437, "Unfortunately, many times the nature, the remaining volume, and the storage procedures of the samples, do not allow to do so." No idea what this means, suggest revision.

Following reviewer suggestions, this sentence has been rephrased.

Line 431: Unfortunately, many times the low volume of collected samples do not allow to perform further laboratory tests

Further authors changes:

As suggested, we thoroughly revised the whole manuscript, making some minor adjustments in order to correct some language mistakes and typos.

The section acknowledgments has been rephrased.
